# Non-Coding RNAs Regulate Placental Trophoblast Function and Participate in Recurrent Abortion

**DOI:** 10.3389/fphar.2021.646521

**Published:** 2021-04-22

**Authors:** Xin Chen, Duan-Ying Guo, Tai-Lang Yin, Jing Yang

**Affiliations:** ^1^ Reproductive Medical Center, Renmin Hospital of Wuhan University and Hubei Clinic Research Center for Assisted Reproductive Technology and Embryonic Development, Wuhan, China; ^2^ Department of Gynecology, Longgang District People's Hospital of Shenzhen, Shenzhen, China

**Keywords:** non-coding RNA, recurrent abortion, placental trophoblast, diagnostic biomarker, diagnosis

## Abstract

Recurrent spontaneous abortion (RSA) is a serious pregnancy complication with an increasing clinical incidence. The various causes of recurrent abortion are complicated. Developments in genetics, immunology, and cell biology have identified important roles of non-coding RNAs (ncRNAs) in the occurrence and progress of recurrent abortion. NcRNAs can affect the growth, migration, and invasion of placental trophoblasts by regulating cell processes such as the cell cycle, apoptosis, and epithelial-mesenchymal transformation. Therefore, their abnormal expression might lead to the occurrence and development of RSA. NcRNAs include small nuclear RNA (snRNA), small nucleolar RNA (snoRNA), ribosomal RNA (rRNA), transfer, RNA (tRNA), circular RNA (cRNA), and Piwi-interacting RNA (piRNA). In this review, we discuss recent research that focused on the function and mechanism of microRNAs (miRNAs), long non-coding RNAs (lncRNAs), and circular RNA (circRNA) in regulating placental trophoblasts. The use of ncRNAs as potential diagnostic and predictive biomarkers in RSA is also discussed to provide future research insights.

## Introduction

Recurrent spontaneous abortion (RSA) refers to spontaneous abortion for two or more consecutive times before 20 weeks of pregnancy (Practice Committee of the American Society for Reproductive Medicine, 2020). About 5% of women of childbearing age worldwide experience RSA (Garrido-Gimenez and Alijotas-Reig, 2015). Therefore, determining the etiology, prevention, and treatment of RSA are vital for human reproductive health and survival. Etiologically, the main known causes of RSA are anatomical abnormalities (Salim et al., 2003), endocrine diseases (Arredondo and Noble, 2006), hereditary diseases (Ogasawara et al., 2000), immune diseases (McNamee et al., 2012), infectious diseases (Kasper et al., 2010), and male factors (Kohn et al., 2016). However, in about 50% of patients, the etiology remains unknown, and these patients are considered to suffer from unknown RSA (URSA) (Sugiura-Ogasawara et al., 2014). In the early stages of a normal pregnancy, the correct execution of the various functions of placental trophoblasts affects the survival of embryos directly. After placenta implantation, the cytotrophoblast (CTB) differentiates into the syncytiotrophoblast (STB) and extravillous trophoblasts (EVTs) (Pollheimer et al., 2018). Subsequently, the EVTs invade the maternal uterus, which allows the placenta to be fixed to the uterine wall, and results in the maternal spiral artery being reshaped to provide nutrition for the developing fetus. Errors at any step in this process can lead to placenta-related pathological pregnancies, including RSA (Wu et al., 2020).

An RNA that does not encode a protein is termed a non-coding RNA (ncRNA). NcRNAs include circular RNAs (circRNAs), long non-coding RNAs (lncRNAs), and microRNAs (miRNA). Despite not encoding proteins, ncRNAs perform important biological functions at the RNA level. For example, they can regulate a variety of important life activities by participating in chromosome remodeling, gene transcription, and post-transcriptional modification (Zhu et al., 2021). MicroRNAs are approximately 20–24 nucleotides in length, and are mainly involved in post-transcriptional regulation. They completely or incompletely bind to the 3′ untranslated region (UTR) of the target mRNA, leading to inhibition of translation or mRNA degradation (Ambros, 2004; Carthew and Sontheimer, 2009). LncRNA refers to an RNA that is more than 200 bp long but does not encode a protein (Mercer et al., 2009; Marchese et al., 2017). LncRNAs have a variety of important biological functions, in which they bind directly to specific DNA, RNA, and protein molecules to affect their transcription, splicing, or translation. LncRNAs can also recruit RNA and proteins in the cytoplasm or nucleus to form functional complexes (Quinn and Chang, 2016). CircRNAs are circular endogenous non-coding RNA molecules without a 5′ cap and 3′ poly (A) tail that are formed by reverse splicing (Zhang et al., 2020). Initially, scholars thought that circRNAs were “junk products” in gene expression; however, advances in DNA and RNA sequencing technology and the development of bioinformatic tools have revealed that circRNAs play important roles in life activities (Hsu and Coca-Prados, 1979; Kristensen et al., 2018). CircRNAs contain a variety of miRNA binding sites, allowing them to act miRNA sponges, by which they act as competing endogenous RNAs (ceRNAs) to ameliorate the miRNA-induced inhibition of target genes, thus enhancing their expression level; and by interacting with disease-related miRNAs, circRNAs play an important regulatory role in the occurrence and development of diseases (Hsu and Coca-Prados, 1979; Kristensen et al., 2018).

In this review, we summarize the role and potential mechanism of ncRNAs in regulating placental trophoblasts, and discuss the latest information about ncRNAs in patients with RSA to further understand their role in RSA.

## Overview of ncRNAs

### MicroRNAs

MiRNAs are highly conserved and participate in almost all pathological and physiological bodily processes, including cell proliferation, growth, development, differentiation, and apoptosis (Bartel, 2004). The first miRNA, lin-4, was identified in 1993 in *Caenorhabditis elegans* (Lee et al., 1993), which paved the way for further research into miRNAs. Lin-4 regulates the expression of lin-14 mRNA negatively by binding to its 3′ UTR, resulting in a decrease in the level of the lin-14 protein. At the same time, the loss of function caused by lin-4 mutation was consistent with the effect caused by mutation of the gene encoding lin-14, which led to a disorder of worm development. Therefore, it was speculated that lin-4 can regulate stages of embryonic development (Wightman et al., 1993). Subsequently, the researchers found a large number of similar endogenous non-coding, single-stranded RNA, composed of 19,023 nucleotides, collectively referred to as miRNAs(Dong et al., 2013). To date, more than 1000 kinds of miRNA have been found in the human body, representing a class of powerful gene regulators. MiRNAs can bind to the mRNA of its target downstream gene and affect the stability and transcription of the targeted mRNA. In mammals, miRNAs affect approximately 60% of protein-coding genes (Griffiths-Jones et al., 2008; Friedman et al., 2009; Kozomara et al., 2019; Wang et al., 2020).

### LncRNAs

LncRNAs are similar to mRNAs in terms of their structure, and are longer than 200 nt (Schmitt and Chang, 2016; Pan et al., 2020). LncRNAs have a complex secondary or tertiary structure and do not show high sequence conservation. LncRNAs can be transcribed from any part of the genome, similar to mRNA, and have a 5′ cap structure and a 3′ poly-A tail structure; however, their coding region is short or non-existent, and they are expressed at low levels in cells (Derrien et al., 2012; Huarte, 2015). The GENCODE database (version 29) shows that there are 19,940 protein coding genes, 16,066 lncRNA genes, and 29,566 lncRNA transcripts in the human genome, and the number of identified lncRNA genes is still increasing (Hadjicharalambous and Lindsay, 2019). In organisms, lncRNAs are expressed widely, functioning in a variety of vital biological activities, such as intracellular signal transduction, chromatin modification, and genomic imprinting (Engreitz et al., 2016).

### CircRNAs

CircRNA are connected to the upstream shear acceptor site through the downstream splicing donor site, and reverse splicing is carried out to form a covalently closed continuous loop (Seimiya et al., 2020). CircRNAs in the cytoplasm of eukaryotic cells were observed using electron microscopy in 1979, and were subsequently found as a pathogenic RNA infection in higher plants (Hsu and Coca-Prados, 1979). Initially, circRNAs were believed to be by-products of splicing (Guo et al., 2014). However, further in-depth study of circRNAs revealed thousands of them in eukaryotic transcriptomes, such as those of human, mouse, nematode, and yeast (Wang et al., 2014). CircRNAs are widely distributed in blood, urine, amniotic fluid, tissues, and organs (Kirby et al., 2019; Vo et al., 2019). In contrast to linear RNA, a circRNA is a closed cyclic molecule without a 5′ cap or 3′ poly (A) tail, making them difficult to degrade by RNA exonuclease and branching enzymes, and providing them with a relatively long half-life compared with linear RNA (Hanan et al., 2017). In addition, researchers identified differences in the types and levels of circRNA expression in different developmental stages of the same tissues and organs, and among different tissues and organs (Hanan et al., 2017). The biological functions of circRNAs have been studied widely. They act as miRNA sponges to regulate the function of miRNAs(Piwecka et al., 2017), as transcriptional or translational regulators to affect protein expression (Memczak et al., 2013; Li C.-H. et al., 2017), and can interact with proteins to regulate gene expression (Du et al., 2017; Dong et al., 2019); surprisingly, some of them also have the potential to encode proteins (Pamudurti et al., 2017).

## Effect of ncRNAs on the Function of Placental Trophoblasts

### Overview of Early Placental Development and Spiral Artery Remodeling

**FIGURE 1:**
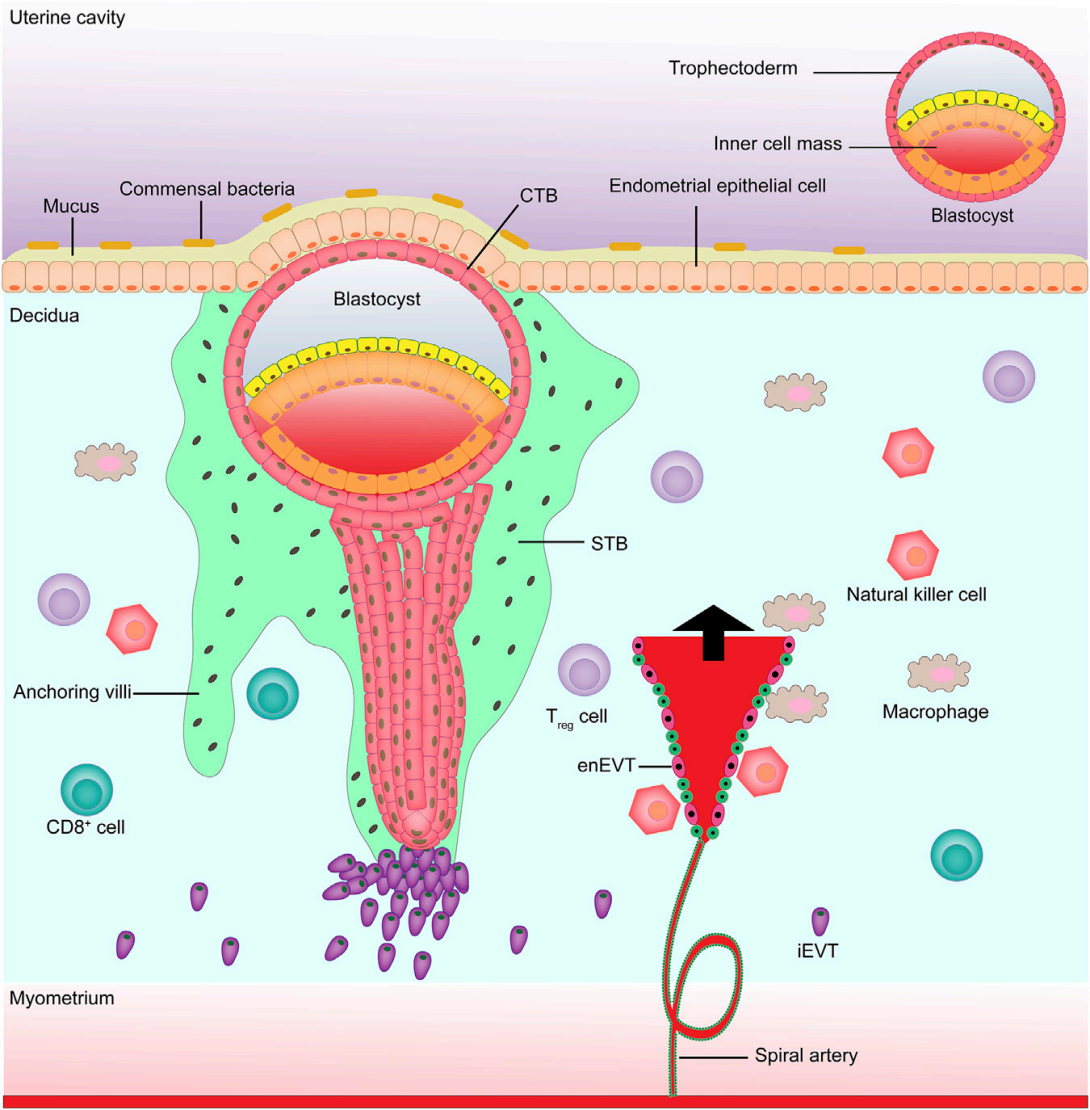
**Maternal-fetal boundary in early pregnancy.** EnEVT, intravascular extravillous trophoblast; iEVT, interstitial extravillous trophoblast; CTB, cytotrophoblast; STB, syncytiotrophoblast.

The placenta is an important organ between the fetus and the mother that plays an important role in the growth and development of the fetus, such as transporting nutrients and metabolic wastes, uric acid, urea, and gas exchange. The placenta also has the biological functions of hormone secretion (e.g., human chorionic gonadotropin) and immune defense, which are closely associated with trophoblast cells (Baines and Renaud, 2017; Chatuphonprasert et al., 2018; Martínez-Razo et al., 2021). The precursor of all trophoblast cells is the trophoblast, which forms the outer layer of the human blastocyst, with fertilization as the time axis. At about 4–5 days after fertilization, the human blastocyst forms and the trophectoderm is separated from the inner cell mass (Knöfler et al., 2019). At about 6–7 days after fertilization, the interaction between the trophectoderm adjacent to the endometrial stroma and the uterine lumen epithelium leads to implantation, at which time the first step in placental development begins (Ali et al., 2020). On the 8th day after fertilization, the trophectoderm in contact with the uterine epithelium is transformed into the highly proliferative cytotrophoblast (CTB) and multinucleated syncytiotrophoblast (STB) (Pötgens et al., 2002; James et al., 2012; Gamage et al., 2016; Boss et al., 2018); at about the 10th day of fertilization, the proliferated CTBs pass through the expanding STBs, resulting in the formation of villi (Prakobphol et al., 2006). At about the 15th day after fertilization, the distal STBs continue to expand to form a trophoblast shell, i.e., anchored villi (Vicovac et al., 1995; Burton and Jauniaux, 2017). Some CTBs anchor the tip of the villi to destroy the covering layer of STBs, invade the uterine stroma, and are transformed into extravillous trophoblasts (EVTs) (Adu-Gyamfi et al., 2020). On the 16th day after pregnancy, two different EVTs appear, i.e., once the detached CTBs come into contact with the decidual extracellular matrix, they differentiate into interstitial extravillous trophoblast cells (iEVTs) (Kemp et al., 2002), which reach the vascular lumen and differentiate into intravascular extravillous trophoblast cells (enEVTs) (Anin et al., 2004; Espinoza et al., 2006). The invasion and migration of EVTs to the maternal spiral artery is another key step in the development of the human placenta, namely spiral artery remodeling (Lyall et al., 2001; Pijnenborg et al., 2006). In early pregnancy, natural killer cells and macrophages surround the spiral artery, while iEVTs are recruited by natural killer cells and macrophages to replace vascular endothelial cells in the spiral artery to initiate the remodeling process (Smith et al., 2009; Wallace et al., 2012) (Figure 1). Understanding the mechanism of ncRNA regulation of abnormal trophoblast function might help to find new treatments for placenta-derived diseases. Below we summarize the role of microRNAs, long non-coding RNAs (lncRNAs), and circular RNAs (circRNAs) in regulating placental trophoblasts and their potential mechanisms (Table 1).

**TABLE 1:** Effect of ncRNAs on the function of placental trophoblasts.

NcRNA	The function of trophoblast	References
lncRNA *NEAT1*	Overexpression of *NEAT1* inhibits the proliferation, migration, invasion, and colony formation of trophoblast cells, and promotes apoptosis	Teng et al. (2020)
miR-181a-5p, miR-378, miR-663, miR-483-3p, miR-514, miR-181a-3, miR-892, miR-34c, and miR-454	Affect the proliferation of trophoblast cells	Arthurs et al. (2019)
circPAPPA	Knockout of circPAPPA results in reduced proliferation and invasion of HTR8-S/Vneo trophoblast cells	Zhou et al. (2019)
MicroRNALet-7a	Inhibition of tumorigenicity and enhancement of apoptosis of JEG-3 cells	Zha et al. (2020)
MiR-124–3p	Inhibits the invasion and migration of trophoblast cells and promotes apoptosis partly through the PLGF-ROS pathway	Tao et al. (2020)
MiR-193b	Targets *IGFBP5* to inhibit autophagy and apoptosis of trophoblasts induced by high glucose	Ji et al. (2020)
miR-133	Affects the apoptosis of trophoblasts in placental tissue	Zhang WM et al., 2019
MiR-125b	Targets *MCL1* to induce apoptosis of HTR8/SVneo cells	Gu et al. (2019)
MiR-200c	Regulation of placental trophoblast apoptosis in rats with preeclampsia	Zhang X. et al. (2019)
MiR-183	Inhibition of trophoblast migration and invasion	Lai and Yu. (2020)
MiR-125b	Regulation of migration and invasion of extravillous trophoblast cells	Tang et al. (2021)
MiR-215–5p	Reduces the ability of trophoblast to migrate and invade	Yang and Meng. (2020)
miR-181b-5p	Regulation of trophoblast migration and invasion	Miao et al. (2020)
MiR-384	Regulation of proliferation and migration of trophoblast cells	Zhou et al. (2020)
MicroRNA-125b	Inhibits the invasion of cytotrophoblasts and damages endothelial cell function	Li Q et al. (2020)
miRNA-29b	Inhibit the growth and migration of trophoblasts	Sun et al. (2020)
LncRNA HOTAIR	Inhibits trophoblast proliferation, migration and invasion	Zhao et al. (2020)
CircTRNC18	Inhibition of trophoblast cell migration and epithelial-mesenchymal transformation	Shen et al. (2019)
lncRNA *H19*	Regulation of angiogenesis of EVTs	Zeng et al. (2020)
lncRNA *TUG1*	Promotes trophoblast proliferation, invasion, and angiogenesis, and inhibits apoptosis	Li et al. (2019)

### Trophoblast Proliferation and Apoptosis

Trophoblast proliferation is a key factor in the normal growth of the placenta. Studies have found that many ncRNAs regulate the proliferation of trophoblasts. For example (Teng et al., 2020),showed that long non-coding RNA nucleus-rich transcript 1 (*NEAT1*) could inhibit trophoblast proliferation in preeclampsia rats through the microRNA-373/Fms related receptor tyrosine kinase 1 (FLT1) axis. Arthurs et al. (Arthurs et al., 2019) pointed out that microRNA mimics targeting the placental renin-angiotensin system could inhibit the proliferation of trophoblast cells (Zhou et al., 2019).found that downregulating the expression of circPAPPA inhibited trophoblast invasion and proliferation through miR-384/signal transducer and activator of transcription 3 (STAT3) pathway.

Trophoblast apoptosis is another key factor in normal placental development. In placental diseases such as preeclampsia (PE) and intrauterine growth restriction (IUGR), an increase in trophoblast apoptosis is the significant pathophysiological feature (Zha et al., 2020). hypothesized that trophoblasts’ biological functions are regulated by let-7a; therefore, they investigated its mechanism in the progress of early-onset severe PE. This led to the identification of the presumptive target genes of let-7a, *BCL2L1* (encoding BCL2 like 1, also known as BCL-XL) and YAP1 (encoding Yes1 associated transcriptional regulator). It was found that let-7a could inhibit *BCL2L1* and *YAP1* expression in trophoblasts (Tao et al., 2020).studied the induction of apoptosis of trophoblasts in early-onset severe PE, and found that miR-124–3p promoted trophoblast apoptosis by targeting placental growth factor. In women suffering from gestational diabetes mellitus, Ji et al. (Ji et al., 2020) detected miR-193b expression, and then simulated the diabetic environment *in vitro* by culturing human trophoblasts in high glucose medium. They then investigated the effects of miR-193b on apoptosis and autophagy in the simulated diabetic environment. The results showed that miR-193b inhibited the apoptosis and autophagy of diabetic trophoblasts by targeting *IGFBP5* (encoding insulin like growth factor binding protein 5) (Zhang WM. et al., 2019). showed that miR-133 participates in the development and process of PE through the Rhodopsin/Rho associated coiled-coil containing protein kinase 1 (ROCK) signaling pathway, which might affect the apoptosis of trophoblasts in placental tissue (Gu et al., 2019).determined the apoptotic effects of miR-125b on HTR-8/SVneo cells *in vitro*. The results showed that in HTR-8/SVneo cells, the expression and translation of the mRNA of miR-125b’s target gene *MCL1* (encoding myeloid cell leukemia 1) were inhibited. In addition, overexpression of miR-125b induced trophoblast cell apoptosis in HTR-8/SVneo cell (Zhang X. et al., 2019). found that in preeclampsia rats, placental trophoblast apoptosis was regulated by miR-200c via the Wnt/β-catenin signal pathway.

Through abundant *in vitro* and *in vivo* experiments, researchers have identified the regulatory mechanisms of ncRNAs, on trophoblast proliferation and apoptosis during embryonic development, and confirmed that ncRNAs play an important role in regulating trophoblast proliferation and apoptosis.

### Epithelial to Mesenchyme transition(EMT), Invasion, and Metastasis

EMT refers to the loss of cell junction and polarity of epithelial cells, which then acquire the phenotypic characteristics of stromal cells, such as decreased adhesion and enhanced migration ability (Horikawa et al., 2017). EVT cell migration and invasion of the decidua and myometrium is an indispensable event in a series of processes from embryo implantation to development. First, at the maternal-fetal interface, mature blastocysts will adhere to decidual tissue, and EVTs will undergo EMT and then invade the endometrial matrix, finally completing embryo implantation (Shu et al., 2020). Then, during implantation and placental development, EVTs, which invade the endometrium, begin to reshape the uterine spiral artery and promote the formation of the blood vessels of the placental bed and the development of the embryo (Pijnenborg et al., 1983; Hustin et al., 1990; Romero et al., 2011; Liu H.-N. et al., 2020). Notably, trophoblast migration and invasion are regulated by ncRNAs (Lai and Yu, 2020). found that increased miR-183 expression could impair the migration and invasiveness of trophoblasts by downregulating the expression of *FOXP1* (Forkhead box P1) and GNG7 (G protein subunit gamma 7) during preeclampsia (Tang et al., 2021). showed that miR-125b could regulate the migration and invasion of extravillous trophoblast cells through the STAT3 signaling pathway and participate in the occurrence of PE (Yang and Meng, 2020). compared the expression level of miR-215–5p and the assumed target gene *CDC6* (cell division cycle 6) in the placenta of 30 patients with PE and 30 women with normal pregnancies. MiR-215–5p inhibited trophoblast migration and invasion by regulating *CDC6* in PE (Miao et al., 2020). found that trophoblast migration and invasion was inhibited in many abnormal events related to trophoblast invasion via miR-181b-5p targeting *S1PR1* (sphingosine-1-phosphate receptor 1) (Zhou et al., 2020). showed that trophoblast proliferation and migration was inhibited by miR-384 targeting of *PTBP3* (polypyrimidine tract binding protein 3) (Li C. et al., 2020).analyzed PE-associated miRNA expression patterns in plasma and identified disordered expression of 16 miRNA in patients with PE. In PE, the expression of hsa-miR-125b in circulation was upregulated abnormally during early pregnancy, but decreased significantly after delivery. The underlying mechanism was discovered to be miR-125b targeting of *KCNA1* (potassium voltage-gated channel subfamily A member 1), which inhibited human trophoblast invasion. In addition downregulation of miRNA-29b in the placenta was observed during gestational diabetes, which might change placental development by regulating trophoblast migration and invasion (Sun et al., 2020). Zhao et al. (Zhao et al., 2020) found that high levels of lncRNA *HOTAIR* inhibited the proliferation, migration, and invasion of trophoblasts by targeting miR-106 in an enhancer of zeste 2 polycomb repressive complex 2 subunit (EZH2)-dependent manner. In addition (Shen et al., 2019), showed that CircTRNC18 inhibits trophoblast cell migration and epithelial-mesenchymal transformation by regulating the miR-762/grainyhead like transcription factor 2 (GRHL2) pathway of preeclampsia.

Thus, we concluded that ncRNAs can affect trophoblast cell migration and invasion through various signaling pathways, thus participating in the occurrence and development of placental abnormality-related diseases.

### Placental Angiogenesis

Adequate blood vessels at the fetal-maternal interface facilitate the transport of nutrients and oxygen from the mother to the embryo, thus ensuring the establishment and maintenance of early pregnancy (Torry et al., 2007). Many studies have shown that abnormal angiogenesis at the maternal-fetal interface might lead to pregnancy complications such as RSA (Banerjee et al., 2013; Ishii et al., 2014). There is limited direct evidence of the involvement of ncRNAs in placental vascular and spiral artery remodeling; however, some scholars have suggested that ncRNAs might regulate placental angiogenesis. For example, researchers examined the clinical samples of pregnant patients (Zeng et al., 2020) and found that lncRNA *H19* was highly expressed in human trophoblasts of early pregnancy, and could regulate the angiogenic ability of extravillous trophoblasts through the H19/miR-106a-5p/vascular endothelial growth factor A (VEGFA) axis (Li et al., 2019). found that lncRNA *TUG1* could target miR-29b to regulate angiogenesis, invasion, apoptosis, and proliferation of trophoblast cells. In addition (Hu and Zhang, 2019), outlined how the abnormal expression of miRNAs in PE and IUGR affects trophoblast infiltration and uterine placental vascular adaptation gene expression; therefore, that article will not be described in detail.

## NcRNAs and RSA

### Overview of RSA

In the past, three or more consecutive miscarriages in a couple before 20 weeks of pregnancy were considered as RSA (Regan, 1991; Kolte et al., 2015). Studies have shown that women who have two consecutive miscarriages are more than 50% likely to have another miscarriage; therefore, some scholars believe that two consecutive abortions can be defined as recurrent abortion (Practice Committee of the American Society for Reproductive Medicine 2012). Generally, RSA has an incidence of about 5%; however, the incidence is increasing (Garrido-Gimenez and Alijotas-Reig, 2015). The etiology of more than half of the cases of RSA is still unexplained (Sugiura-Ogasawara et al., 2014).

**FIGURE 2:**
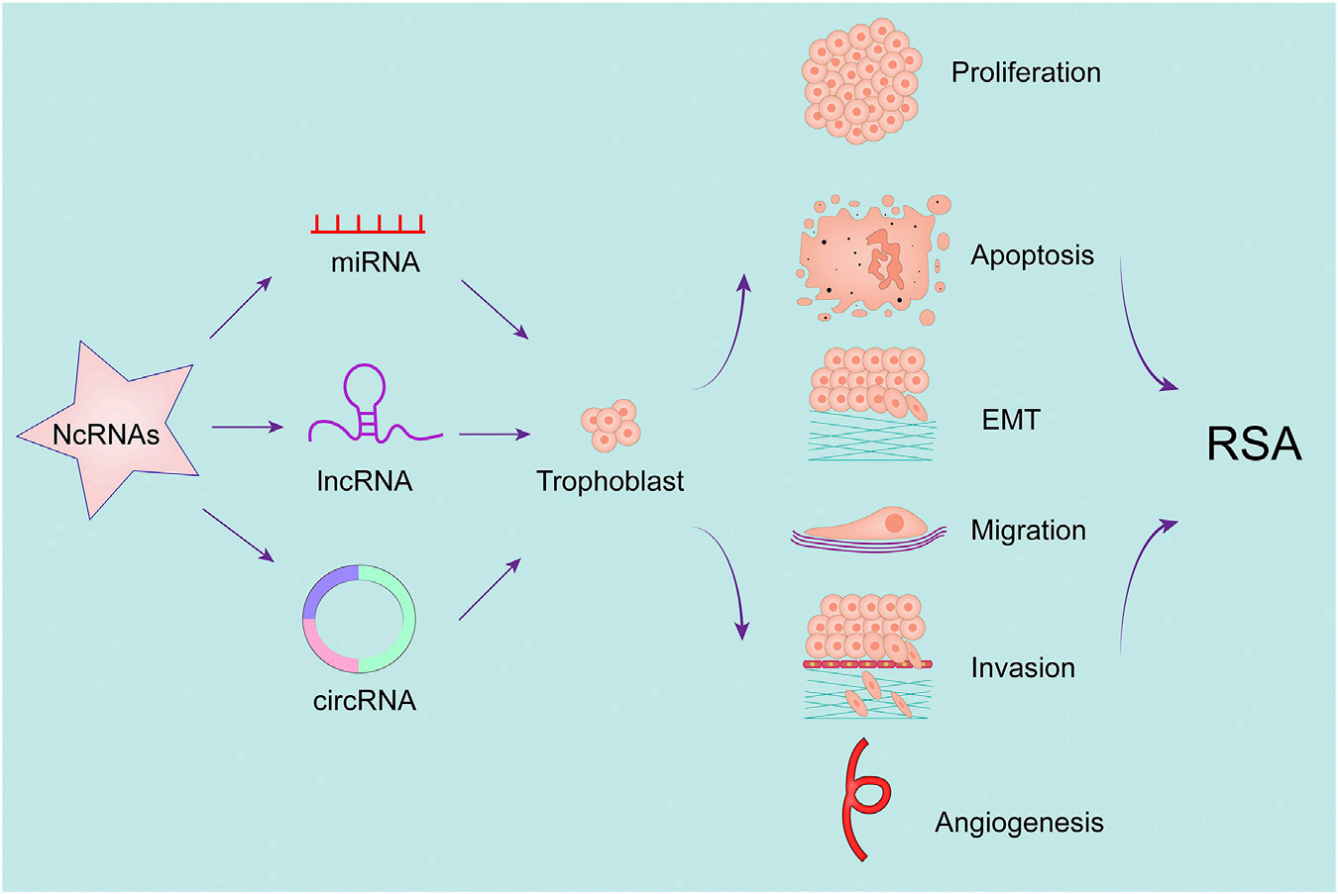
NcRNA regulate placental trophoblast function and participate in recurrent abortion.

Below we briefly describe a number of studies of ncRNAs in RSA (Figure 2 and Table 2).

**TABLE 2:** NcRNAs related to the pathogenesis of RSA.

NcRNA	Expression in RSA	Model (***in vivo***, ***in vitro*, human)**	Regulation of trophoblast	References
MiR-93	Upregulation	*In vitro*, human	Proliferation, migration, invasion and apoptosis	Liu X. et al. (2020)
miR-19b	Upregulation	*In vitro*, human	Apoptosis	Tian et al. (2020)
miR-494	Downregulation			
miR-27a-3p	Upregulation	*In vitro*, human	EMT, migration and invasion	Ding et al. (2019)
miRNA-365	Upregulation	*In vitro*, human	Apoptosis	Zhao et al. (2017)
miR-520	Upregulation	*In vitro*, human	Apoptosis	Dong et al. (2017)
MicroRNA-16	Upregulation	*In vivo*, *in vitro*, human	Angiogenesis	Zhu et al. (2016)
lncRNA *PVT1*	Downregulation	*In vitro*, human	Proliferation, migration, invasion and apoptosis	Yang et al. (2020)
lncRNA *MALAT1*	Downregulation	*In vitro*, human	Proliferation, migration, invasion, apoptosis and angiogenesis	Wang et al. (2019), Wang et al. (2018)
lncRNA *SNHG7-1*	Downregulation	*In vitro*, human	Proliferation, migration, invasion and apoptosis	Xiang et al. (2019)
circ-ZUFSP	Downregulation	*In vivo* and *in vitro*	Migrate and invade	Li Z. et al. (2020)

### MiRNAs and RSA

Previously, researchers pointed out that the expression profile of miRNAs in chorionic villi might be related to RSA (Tang et al., 2016). The expression of miR-93 in clinical samples was significantly increased in the chorionic villi of patients with RSA. The upregulation of miR-93 inhibited the proliferation, migration and invasiveness of human trophoblast HTR-8/SVneo cells, and promoted apoptosis *in vitro*. By contrast, the downregulation of miR-93 reversed these effects (Liu H.-N. et al., 2020). (Tian et al., 2020) investigated the potential regulatory relationship of *PTEN* (phosphatase and tensin homolog) and miRNAs in the placental villi of patients with RSA. The results showed that the overexpression of *PTEN* plays an important role in the pathogenesis of RSA, and the synergistic effect of miR-19b and miR-494 regulates *PTEN* directly. These ncRNAs are involved in the abnormal role of villi (Ding et al., 2019). analyzed the expression of USP25 (ubiquitin specific peptidase 25) in the placental villi of patients with RSA, and then evaluated the role of USP25 in the invasion and migration of trophoblast EMT. In addition, the effects of miRNAs on *USP25* expression were explored using luciferase reporter gene analysis and bioinformatic prediction. In trophoblasts, *USP25* expression was evaluated after transfection with microRNA mimics or inhibitors. The miR-27a-3p/USP25 axis was observed to inhibit trophoblast migration and invasion in the pathogenesis of RSA (Zhao et al., 2017). pointed out a new mechanism whereby miRNA-365 regulates trophoblast apoptosis in RSA. Another studies showed that miR-520 can promote trophoblast apoptosis induced by DNA damage by targeting *PARP1* (poly (ADP-ribose) polymerase 1), thus participating in the occurrence and development of RSA (Dong et al., 2017). In addition, miR-16 regulates placental angiogenesis and development by targeting the expression of vascular endothelial growth factor (VEGF), and participates in the pathogenesis of RSA (Zhu et al., 2016). (Wang et al., 2016)determined the miRNA expression profile in the decidua or villi using deep sequencing analysis, which indicated that the pathogenic process of RSA might be related to changes in the miRNA expression profile in the decidua and villi.

### LncRNAs and RSA

LncRNAs have been noted as important regulators of a variety of cellular processes, including pregnancy (Kung et al., 2013; Bouckenheimer et al., 2016). Previously, researchers identified 1449 differentially expressed lncRNAs from chorionic villi of patients with recurrent miscarriage (RM) patients, providing evidence that lncRNAs could participate in the physiological and pathogenic pathways of human RM(Wang et al., 2017). Yang et al. (Yang et al., 2020) studied the transcriptional regulation of lncRNA *PVT1* and its effects on the biological behavior of trophoblasts, which might be related to the pathogenesis of RSA. A study (Wang et al., 2019)found that the levels of *NEAT1* and metastasis associated lung adenocarcinoma transcript 1 (MALAT1) in tissue samples of patients with RSA were significantly decreased, and knockdown of the *MALAT1* gene could lead to a decrease in proliferation and an increase in apoptosis of trophoblasts and primary chorionic trophoblasts.

LncRNAs can regulate the transcription and expression of downstream genes by targeting miRNAs, thus promoting disease development (Li Z. et al., 2017; Ling et al., 2017). For example, the level of *MALAT1* in chorionic villi of 36 patients with RSA decreased, and it was found that *MALAT1* interacts directly with an miRNA (Wang et al., 2018). Subsequent functional experiments showed that *MALAT1* regulates cell invasion, migration, apoptosis, and proliferation through direct interactions with miR-375, miR-205, miR-15, and miR-383, which might lead to the pathogenesis of RSA. Xiang et al. (Xiang et al., 2019) found that the level of *SNHG7* (small nucleolar RNA host gene 7) in RSA villi decreased, and it could cause RSA by regulating miR-34a to inhibit the proliferation and invasion of trophoblast cells.

### CircRNAs and RSA

To date, there have been few studies on circRNAs and RSA. Li et al. found that compared with women with normal pregnancies, 123 differentially expressed circRNAs were found in patients with early RSA, including 78 upregulated and 45 downregulated circRNAs (Li Q. et al., 2020). Another study investigated the effects of circ-ZUFSP on trophoblast function by overexpressing and downregulating circ-ZUFSP *in vitro*, which demonstrated the molecular mechanism of circ-ZUFSP regulation of trophoblast migration and invasion, and provided new indicators to diagnose and treat RSA (Li Z. et al., 2020).

## NcRNAs as Diagnostic Biomarkers and Potential Therapeutic Targets for RSA

Disease biomarkers should be highly specific and sensitive, exist stably in the circulatory system, and their acquisition should be inexpensive and fast. At present, few molecules meet these criteria. The clinical prediction of RSA is limited to low-specificity biomarkers, such as antiphospholipid antibodies (Balasch et al., 1996) and progesterone (Jordan et al., 1994). The circulatory system is rich in ncRNAs, such as miRNA, lncRNA, and cirRNA, which are either secreted actively as acellular circulating RNA or are released passively released from tissue or via cell injury. The ncRNAs in these circulatory systems are quite unstable, so they usually bind to lipoproteins or are wrapped by exocrine bodies to avoid denaturation (Barth et al., 2020). Increasing evidence shows that ncRNA, such as miRNAs, lncRNAs, and circRNAs in the circulatory system of patients with RSA are promising biomarkers for early diagnosis and treatment. Moreover, circulating ncRNAs might also play an important role in the development and pathogenesis of RSA. Consequently, identifying and evaluating potential circulating biomarkers for RSA will contribute to the diagnosis and prevention of RSA. In this review, we summarized several studies on the potential role of ncRNAs as a plasma and serum biomarkers of RSA.

As mentioned earlier, ncRNAs play an important role in the occurrence and development of RSA (such as trophoblast proliferation and apoptosis, EMT, invasion and metastasis, and placental angiogenesis). Therefore, ncRNAs could be regarded as diagnostic markers and therapeutic targets for RSA. In different diseases, ncRNAs can be detected in the tissue, blood, and urine of patients, and their levels plays an important role in the early diagnosis and late prognosis of the disease (Adachi et al., 2010; Zhang et al., 2010). Notably, miRNAs usually exists in peripheral blood in a more stable form than traditional biomarkers (Vasilescu et al., 2009; Wang et al., 2010). LncRNAs are relatively more resistant to endogenous ribonucleases, which makes them more stable in the blood (Tong et al., 2015). In addition, the high abundance, diversity, structural stability, and tissue specificity of circRNAs also make them more persistent in the circulatory system or body fluids (Memczak et al., 2013; Salzman et al., 2013). Therefore, ncRNAs are generally more stable and representative than traditional biomarkers. Based on this characteristic, ncRNAs might emerge as ideal diagnostic clinical biomarkers and therapeutic targets in RSA. Qin et al. (Qin et al., 2016) used gene microarrays and real-time quantitative reverse transcriptase polymerase chain reaction (qRT-PCR) to analyze the difference in miRNA expression between plasma samples from patients with RSA and from women with normal pregnancy (NP), and found that four circulating miRNA (miR-320b, miR-146b-5p, miR-221–3p, miR-559) were upregulated and one circulating miRNA (miR-101–3p) was downregulated. This suggested that circulating miRNAs might be involved in the pathogenesis of RSA and could become new biomarkers for RSA diagnosis. Coincidentally, some researchers (Yang et al., 2018)verified the expression of miRNAs (miR-23a-3p, 27a-3p, 29a-3p, 100–5p, 127–3p, and 486–5p) in the peripheral plasma and serum of women with RSA and NPs using qRT-PCR. The results showed that compared with those in women with NPs, the levels of miR-127–3p, miR-100–5p, miR-29a-3p, and miR-27a-3p, and in the peripheral blood plasma of women with RSA were significantly higher. The level of miR-486–5p in plasma decreased significantly. In contrast, serum miR-23a-3p and miR-127–3p decreased significantly, while serum miR-486–5p increased significantly. This suggested that circulating levels of these miRNAs might be associated with the pathogenesis of RSA and could represent diagnostic biomarkers for RSA. At the same time, the authors found that in recurrent abortion, the levels of miR-127–3p and miR-486–5p in plasma correlated negatively with the levels of miR-127–3p and miR-486–5p in serum, and speculated that this phenomenon was caused by the different sources of these miRNAs. However, we disagree with this speculation and think that phenomenon is more likely to be caused by individual differences; therefore at present, more research is required to investigate the role of miRNAs as biomarkers of the increased risk of recurrent spontaneous abortion. In conclusion, miRNAs in circulation as biomarkers of the increased risk of recurrent abortion have a good prospect and clinical value; however, more clinical evidence is needed to support their clinical use.

### Restrictions on the use of Non-coding RNA in Clinic

Although research into non-coding RNAs has become a hot topic, it is still a long way from clinical application. Currently, the main limitations for clinical use of non-coding RNA are as follows: 1) ncRNAs are often dynamic in the circulatory system, and the samples collected from patients in a certain period of time only represent the expression status at that time (May et al., 2021). Therefore, continuous dynamic and standardized monitoring is essential if ncRNAs are to be used as biomarkers of increased risk of RSA; however, this will lead to an increase in the cost of treatment. 2) Most of studies of RSA and ncRNAs are still at the stage of *in vitro a*nd animal experiments; therefore, it is difficult to know whether ncRNAs will cause changes in the expression of other genes when used as a targeted therapy, which needs more clinical experimental evidence. 3) The use of RNA to treat diseases often requires a suitable vector transport. Currently, the most commonly used vectors are recombinant viruses (such as adenoviruses and lentiviruses); however, the use of viruses as vectors might lead to the risk of infection in other organs and could trigger immune responses in the body (Dong et al., 2021). The arrival of the CRISPR/Cas9 system hold promise for ncRNA therapy; however, because of potential off-target effects and ethical restrictions that might apply to gene editing technology, further research and exploration are needed to put it into clinical use (Luo et al., 2021). 4) Clinical treatment requires high purity, high stability, and high bioactivity of ncRNA. At present, the main method of RNA synthesis is chemical synthesis, and the purification method is mainly high performance liquid chromatography; however, highly pure and bioactive ncRNA would induce higher costs, which will obviously increase the financial burden on patients. Therefore, it is very important to develop more effective and economical methods for RNA synthesis and purification (Baptista et al., 2021).

## Conclusion

The present review summarized the latest advances in the role, potential clinical application, and potential molecular mechanisms of ncRNAs related to the occurrence and development of RSA. The incidence of RSA in women of childbearing age is increasing, which seriously affects their quality of life and the health of mothers and infants. In addition, it has an impact on parents’ mental health, and might even hinder the reproduction of the whole human population. Therefore, how to improve the pregnancy success rate of patients with RSA has been the focus of clinical research. Intensive research has identified novel molecules, such as immune factors, which have improved the diagnosis, prevention, and treatment of this disease. Recent research has partially clarified the contribution and mechanism of ncRNAs in RSA. However, we still lack a comprehensive understanding of the process, and many issues remain to be discussed. Thus, their inclusion in medical guidelines is still a long way off. Therefore, we suggest that researchers study larger population samples to obtain sufficient evidence-based medicine to prove that ncRNAs are clinically applicable in to diagnose and treat disease of early pregnancy, such as RSA.
